# Raman Imaging of Plant Cell Walls in Sections of *Cucumis sativus*

**DOI:** 10.3390/plants7010007

**Published:** 2018-01-25

**Authors:** Ingrid Zeise, Zsuzsanna Heiner, Sabine Holz, Maike Joester, Carmen Büttner, Janina Kneipp

**Affiliations:** 1 Department of Chemistry, Humboldt-Universität zu Berlin, Brook-Taylor-Str. 2, 12489 Berlin, Germany; ingrid.zeise@chemie.hu-berlin.de (I.Z.); heinerzs@hu-berlin.de (Z.H.); maike.joester@chemie.hu-berlin.de (M.J.); 2 School of Analytical Sciences Adlershof SALSA, Humboldt-Universität zu Berlin, Albert-Einstein-Str. 5-9, 12489 Berlin, Germany; 3 Institute of Agricultural and Horticultural Sciences, Humboldt-Universität zu Berlin, Lentzeallee 55/57, 14195 Berlin, Germany; Sabine.Holz@gmx.net (S.H.); carmen.buettner@agrar.hu-berlin.de (C.B.); 4 BAM Federal Institute for Materials Research and Testing, Richard-Willstatter-Straße 11, 12489 Berlin, Germany

**Keywords:** *Cucumis sativus*, cell wall, xylem, Raman spectroscopy, chemical imaging, cellulose, lignin, principal component analysis, hierarchical cluster analysis, carotene

## Abstract

Raman microspectra combine information on chemical composition of plant tissues with spatial information. The contributions from the building blocks of the cell walls in the Raman spectra of plant tissues can vary in the microscopic sub-structures of the tissue. Here, we discuss the analysis of 55 Raman maps of root, stem, and leaf tissues of *Cucumis sativus*, using different spectral contributions from cellulose and lignin in both univariate and multivariate imaging methods. Imaging based on hierarchical cluster analysis (HCA) and principal component analysis (PCA) indicates different substructures in the xylem cell walls of the different tissues. Using specific signals from the cell wall spectra, analysis of the whole set of different tissue sections based on the Raman images reveals differences in xylem tissue morphology. Due to the specifics of excitation of the Raman spectra in the visible wavelength range (532 nm), which is, e.g., in resonance with carotenoid species, effects of photobleaching and the possibility of exploiting depletion difference spectra for molecular characterization in Raman imaging of plants are discussed. The reported results provide both, specific information on the molecular composition of cucumber tissue Raman spectra, and general directions for future imaging studies in plant tissues.

## 1. Introduction

Raman microspectroscopy gives spatially resolved vibrational information from complex biological samples and provides a very efficient means for the investigation of different plant materials, ranging from pollen specimens [1,2] over fruit [3,4] and roots [5] to different woods [6,7,8,9,10,11]. Thereby, it can serve many different analytical tasks [12,13,14]. In tissue sections, both the molecular composition and supramolecular structure of the cell walls can be studied [8,15]. In the work presented here, we discuss Raman microspectra of sections from different tissues of *Cucumis sativus* (cucumber) plants. While imaging of individual tissue sections can give detailed histological information, including number and size of cells, and thickness of the cell walls, utilization of the spectroscopic information to derive general information on the molecular composition of a tissue requires the analysis of many tissue sections from several plants together. Here, we generated Raman maps of 55 sections that we obtained by matrix free cutting of fresh root, hypocotyl, and leaf tissue, and analyze the morphological information that we get from the Raman chemical and multivariate images. 

As we acquired spectra from a theoretical spot size of about 0.87 µm in diameter, yet in steps of 1 µm, no part of the tissue area is sampled twice, and exposure time per spot is short and the same for all molecules in each sampled spot. This is different from typical oversampling, where step sizes are chosen smaller than the spot size and partial overlap occurs. Irradiation of the same areas in the tissue multiple times in oversampling or multiple measurements of the same spots, or averaging of spectra that were reported to be helpful in improving signal-to-noise-ratios [7,16,17], result in very efficient bleaching of fluorescence or different kinds of photochemical reactions. This was not the case here, and so we find changes in the background of the Raman spectra within the mapping data sets. In addition to the decrease of the autofluorescence, we discuss Raman spectral contributions that are due to the pre-/resonant excitation of pigment molecules that are not washed away in our sample preparation procedure. Molecules such as carotenoid are bleached by excitation in the visible wavelength range and, as a consequence, cannot be analyzed, unless the concept of depletion difference spectra [18,19] is utilized in the imaging procedure.

## 2. Results and Discussion

### 2.1. Histological Information from Chemical Composition

Sections of fresh leafs, stems and roots of four *Cucumis sativus* (cucumber) plants were cut without embedding to prevent contaminations of the sample and investigated by raster scanning Raman microscopy using an excitation wavelength of 532 nm and a collection time of 1 s per spectrum. In total, 55 maps were collected, and ~95,000 spectra were analyzed, further details are provided in Table 1.

**Figure 1:**
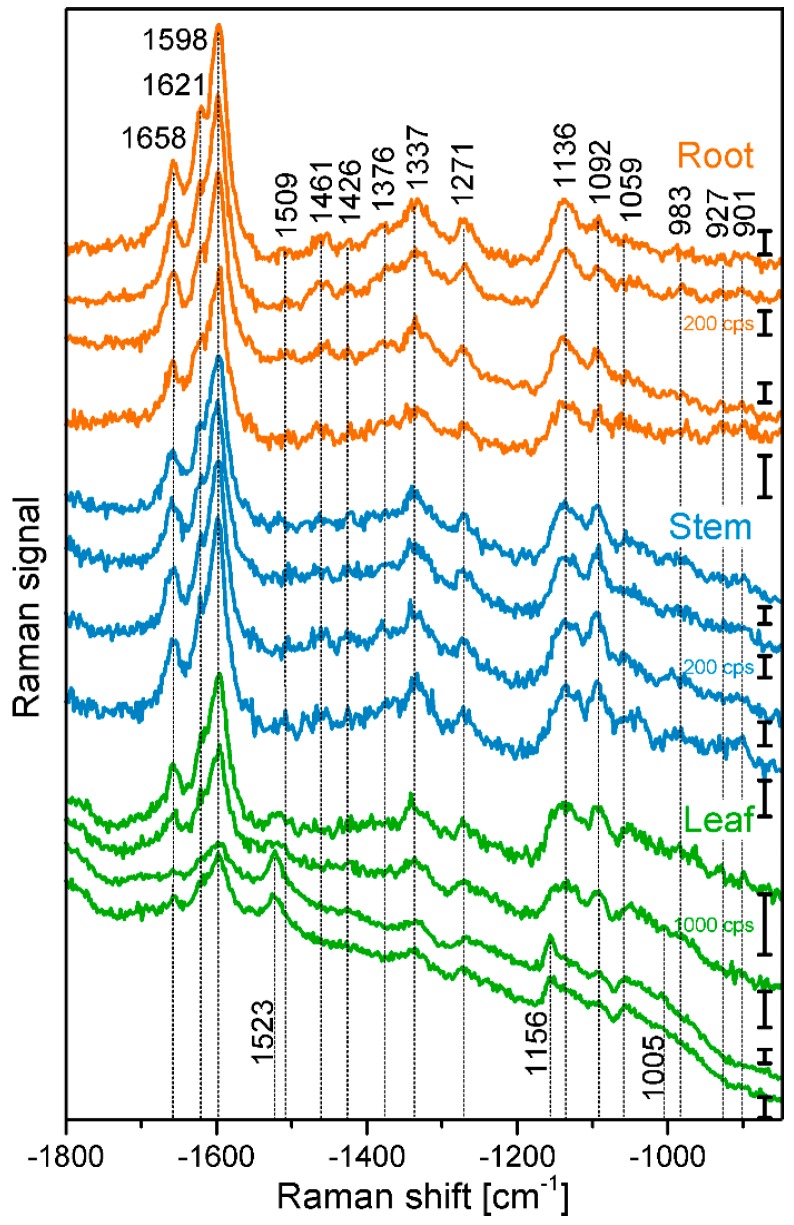
Exemplary spectra from the xylem cell wall regions of root, stem, and leaf sections of cucumber plants, from different anatomical regions: root (top to bottom), cell corner (cc), middle lamella, cc; stem, all secondary cell wall; leaf, all secondary cell wall. Accumulation time: 1 s, excitation wavelength: 532 nm, excitation intensity: 1.7 × 10^6^ W/cm^2^. Spectra are not pre-treated, but stacked for clarity.

Figure 1 shows example spectra that were randomly chosen from the maps of root, stem, and leaf cross sections. The spectra show several signals of lignin and cellulose, assignments of the most prominent bands are given in Table 2. At 1598 cm^−1^, 1621 cm^−1^, and at 1658 cm^−1^, typical signals of lignin appear, caused by different stretching vibrations of the aryl ring of coniferyl aldehyde and coniferyl alcohol. Very strong spectral contributions from lignin have been reported frequently, specifically when excitation at visible wavelengths, e.g., at 532 nm was used. On the one hand, lignin is an important building block of the plant cell wall. On the other hand, although the electronic absorption of lignin is in the UV [20,21], the Raman signal of lignin or of related molecules could benefit from electronic pre-resonance effects that result in strong signals in the region between 1550 cm^−1^ and 1700 cm^−1^.

Cellulose bands are visible, for example at 1092 cm^−1^, assigned to C-C and C-O stretching modes, and at 1337 cm^−1^, due to deformation vibrations (δ(HCC) and δ(HCO)) of the macromolecule. Both the signal of the stretching vibration [15,28,29,30,31], as well as the band of the HCC and HCO deformation vibration [22,24,28] are sensitive to cellulose microfibril orientation.

In the spectra of the leaf sections (Figure 1, bottommost spectra) and in some spectra from the stem samples, carotenoid signals (at 1523 cm^−1^, 1156 cm^−1^, 1005 cm^−1^) are very prominent [3,25,26,27], due to the strong resonance enhancement that is in place at the chosen excitation wavelength of 532 nm. By fast visible inspection, the spectra from the different tissues (Figure 1), specifically those of root and stem look very similar, although relative intensities vary slightly between the tissues, with spectra from leaves being more distinct. Images that are generated from the mapping data provide a quite differentiated view on absolute and relative intensities of the signals that give information on the substructures and morphological properties of the tissues from the different plant organs.

**Figure 2:**
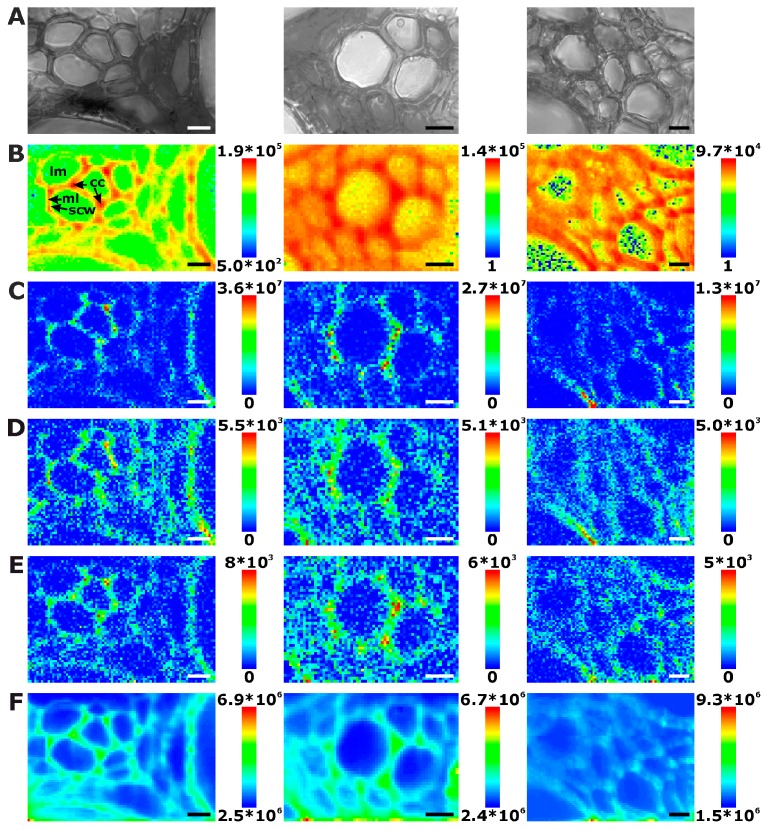
Chemical images of three exemplary mapping data sets of cross sections of cucumber root xylem. (**A**) Bright field images; (**B**) Integral intensity in the region 1550–1700 cm^−1^, obtained after baseline correction; (**C**) Product of the intensities of the cellulose bands at 1092 cm^−1^ and 1337 cm^−1^, respectively, cf. (**D**,**E**); (**D**) Intensity at 1092 cm^−1^ (baseline corrected in the range 1070–1108 cm^−1^); (**E**) Intensity at 1337 cm^−1^ (baseline corrected in the range 1313–1358 cm^−1^); (**F**) Intensity integrated over the full spectral range 600–2000 cm^−1^. Abbreviations: lm: lumen; cc: cell corner; ml: middle lamella; scw: secondary cell wall. Scale bars: 10 µm, mapping step size: 1 µm, excitation wavelength: 532 nm, excitation intensity: 1.7 × 10^6^ W/cm^2^, accumulation time: 1 s.

Figure 2 displays three examples of Raman maps of primary root xylem tissue. In the bright field images, the thick walls of the xylem cells appear dark, while the phloem cell walls look very bright and thin (Figure 2A, e.g., uppermost part). Figure 2B shows chemical maps generated from the absolute intensity of the characteristic Raman signals of lignin between 1550–1700 cm^−1^ (see Table 1 for assignments) [23,24,28,32]. Assuming a similar thickness across each tissue section, the variation in the lignin Raman intensity must reflect a varying local concentration of lignin. Lignification is present all over the xylem of the root sections, even in smaller cells between the larger vessels. In contrast, the cells of the phloem do not show intense lignin signals (Figure 2B). Please note that the color range from blue (for low signals) to red (for high signals) in Figure 2B was used on a logarithmic intensity scale, as this scale provides particular sensitivity for discrimination within areas of low intensities, and hence easy differentiation between high and low lignin concentration rather than within the areas of high lignin content. As is visible in Figure 2B, the corners of the lignified cell walls, as well as the regions where we expect the middle lamella to be located show relatively high amounts of lignin. This observation is similar to the distributions of Raman spectra [10,11,15,33,34] and fluorescence signals of lignin in several kinds of plants [35].

**Figure 3:**
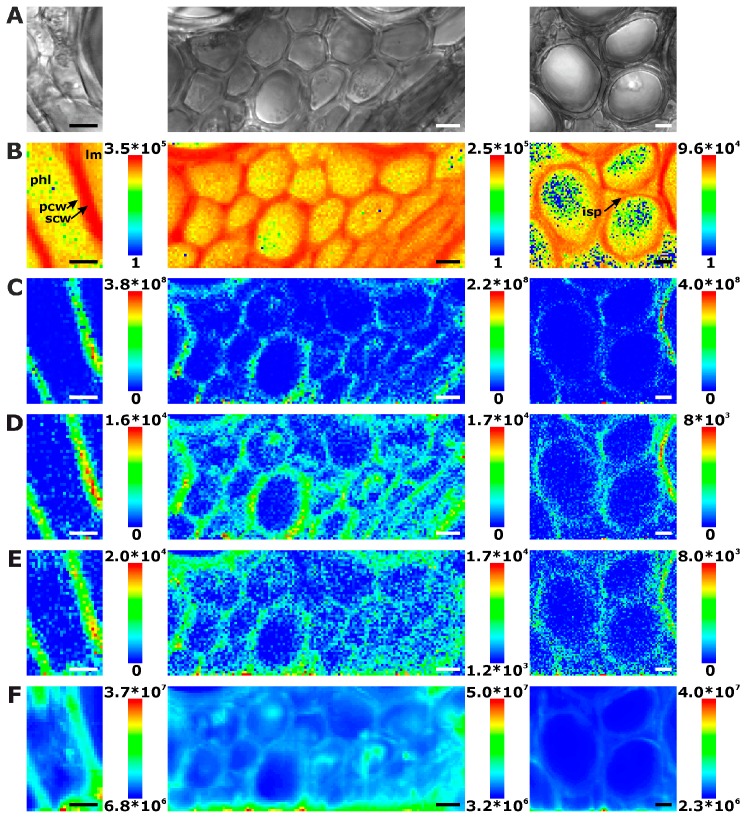
Chemical images of three exemplary mapping data sets of cross sections of cucumber stem xylem. (**A**) Bright field images; (**B**) Integral intensity in the region 1550–1700 cm^−1^, obtained after baseline correction; (**C**) Product of the intensities of the cellulose bands at 1092 cm^−1^ and 1337 cm^−1^, respectively, cf. (**D**,**E**); (**D**) Intensity at 1092 cm^−1^ (baseline corrected in the range 1070–1108 cm^−1^); (**E**) Intensity at 1337 cm^−1^ (baseline corrected in the range 1313–1358 cm^−1^); (**F**) Intensity integrated over the full spectral range 600–2000 cm^−1^. Abbreviations: lm: lumen; phl: phloem; scw: secondary cell wall; pcw: primary cell wall; isp: intercellular space. Scale bars: 10 µm, mapping step size: 1 µm, excitation wavelength: 532 nm, excitation intensity: 1.7 × 10^6^ W/cm^2^, accumulation time: 1 s.

In the xylem vessels of the stem sections, lignin is distributed in a similar fashion (Figure 3B). Here, the most intense signals are found in the regions of the secondary cell walls (Figure 3B, first and last panel), due to the absence of middle lamellae and cell corners. The signals in the regions where the primary and tertiary cell walls can be expected are much lower in intensity than in the secondary cell walls.

Some examples (e.g., Figure 3B middle panel) show most lignin signals in the regions of cell corners and middle lamella, where the xylem cell walls abut on each other. Phloem cell walls, appearing bright and thin in bright field images, are not visible in these maps either (Figure 3A).

**Figure 4:**
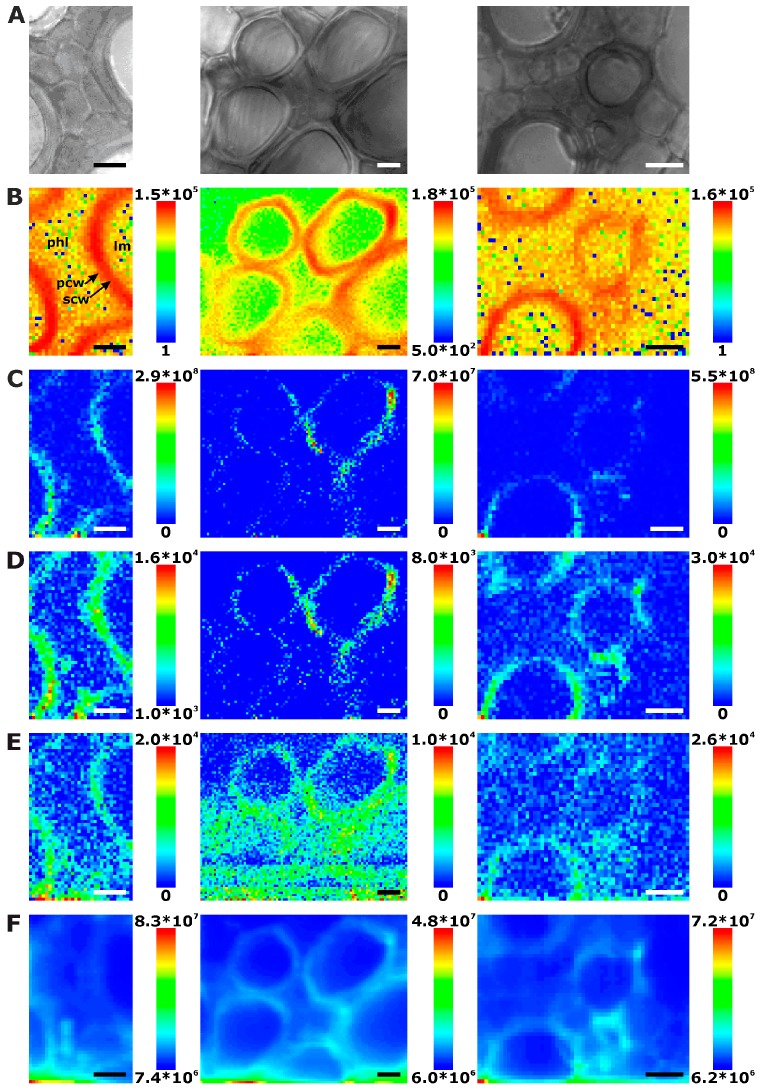
Chemical images of three exemplary mapping data sets of cross sections of cucumber leaf xylem. (**A**) Bright field images; (**B**) Integral intensity in the region 1550–1700 cm^−1^, obtained after baseline correction; (**C**) Product of the intensities of the cellulose bands at 1092 cm^−1^ and 1337 cm^−1^, respectively, cf. (**D**,**E**); (**D**) Intensity at 1092 cm^−1^ (baseline corrected in the range 1070–1108 cm^−1^); (**E**) Intensity at 1337 cm^−1^ (baseline corrected in the range 1313–1358 cm^−1^); (**F**) Intensity integrated over the full spectral range 600–2000 cm^−1^. Abbreviations: lm: lumen; phl: phloem; scw: secondary cell wall; pcw: primary cell wall. Scale bars: 10 µm, mapping step size: 1 µm, excitation wavelength: 532 nm, excitation intensity: 1.7 × 10^6^ W/cm^2^, accumulation time: 1 s.

Because the leaves that were chosen for the experiments were quite young and do not show old, thickened xylem cell walls, only few spectra have intense Raman signals of lignin in the leaf sections (Figure 4B). This is also evident in the example spectra displayed in Figure 1 that show very low relative intensities of the strongest lignin marker bands. As will be discussed below, by multivariate imaging of these data, we do achieve sufficient image contrast based on lignin though. In the leaf sections, lignin is clearly localized in the region of the secondary cell wall (Figure 4B), different from its main accumulation in the regions of the cell corners and middle lamella in the root sections (Figure 2B). Also in leaves, the phloem cell walls, as shown above in the root and stem sections, cannot be imaged using lignin signals due to the absence of lignification. 

Raman chemical mapping of signals characteristic of cellulose provides a further means to image the structure and morphology of the plant cell walls. The Raman spectrum of cellulose is very rich in signals and gives information on both the chemical structure of the macromolecule and on its supramolecular arrangement within in the cell wall [15,28,29,30,31]. Nevertheless, as can be seen in the spectra (e.g., the bottommost spectra of Figure 1), superposition of the cellulose bands with contributions from other molecules in the complex plant material can occur, and intensities of the cellulose bands can be low if material density or concentration is low. To minimize non-specific spectral contributions, maps of cellulose distributions were generated using the product of the cellulose signals at 1092 cm^−1^ and at 1337 cm^−1^ (Figure 2C, Figure 3C, and Figure 4C). The cellulose maps from data sets of root tissue are displayed in Figure 2C. High signals that can be attributed to cellulose can be found in the regions of the cell corners. Cellulose signals are also present in the regions of the secondary cell walls and middle lamellae. In all these structures, lignin signals are present as well. The differential localization of lignin within the substructures of the cell wall, namely high concentrations in the cell corners and middle lamellae that was reported in other studies [7,8], is not found here, most likely due to the chosen lateral resolution in the raster scan.

As can be seen by comparison of the signal product maps in Figure 2C with the maps generated from the individual intensities of the two vibrations, the C–C and C–O stretching band at 1092 cm^−1^ (Figure 2D) and the HCC and HCO deformation vibration at 1337 cm^−1^ (Figure 2E), respectively, improved image contrast enables the identification of the regions of ordered cellulose structures. Specifically in the leave sections, utilization of the band at 1337 cm^−1^ alone (Figure 4E) does not seem useful for mapping of the cellulose distribution, mainly due to the quite low absolute intensities at this position and a stronger background signal (cf. example spectra in Figure 1).

Especially in the product maps (Figure 2C, Figure 3C and Figure 4C), directionality of high intensity areas due to the sensitivity of some cellulose bands towards cellulose microfibril orientation can be observed. In our experiments, the signals from the cellulose microfibrils in a highly ordered cell wall depend on the direction of the cell wall with respect to the (arbitrary, but fixed) polarization of the laser used to excite the Raman scattering. This observation is in agreement with previous reports [15,28,29]. In contrast, the lignin signal (Figure 2B, Figure 3B and Figure 4B) is distributed isotropically throughout the cell walls. Raman microscopy revealing unequally the distributions of cellulose and lignin components due to the high ordering of the first and the unordered arrangement of the latter was supported by combination with other microscopies recently, where similar results were obtained in tissue sections from grasses [15].

Mapping the lignin and cellulose distribution, we obtain morphological information from the spatial distribution of the chemical composition with respect to the main constituents of the cell walls. The maps shown in Figure 2, Figure 3 and Figure 4 are representative for each plant organ. In the stem sections, most xylem cell walls resemble those in Figure 3, with phloem cells or intercellular space between the xylem cells. Nevertheless, some stem xylem regions are strongly thickened, and even thin cell walls are lignified and can be imaged easily, for example in the middle panels of Figure 3. The maps of central veins of the leaf sections of the first true leaf (non-cotyledon) show xylem vessel cell walls that are clearly separated thus without cell corners or middle lamella (Figure 4). 

By an analysis of certain parameters in the spectra from all Raman maps of the three investigated tissues we can draw conclusions on some morphological properties of the cells, including density of the tissues or thickness of the cell walls. Table 1 summarizes some parameters resulting from a statistical analysis of the mapping data. Here, the count of spectra that give a signal in the frequency region from 1550 cm^−1^ to 1800 cm^−1^ that contains the contributions of lignin is used to localize the cell walls. From this, an average portion of cell wall spectra for each map is calculated (Table 1, last column), allowing us to infer on cell density and/or cell wall thickness. Furthermore, the total amount of different cells contained in all data sets can be counted, as well as the amount of cells that were raster scanned completely can be estimated, enabling an estimate of a number of cell wall spectra per sampled cell for each tissue type. The amount of cell wall spectra per sampled cell provides an indirect measure of cell size and/or thickness of the cell walls. Nevertheless, as discussed above, several smaller cells of the phloem in stem and leaf sections did not provide strong signals, and hence are not counted here, while small xylem cells appear more pronounced in the maps of the root sections and increase the number of sampled cells there. This leads to a relatively low amount of cell wall spectra per cell in the data of the root sections. 

The data displayed in Table 1 serve as an example for the possibility to analyze specific histological features, in this case lignified cell walls from many different tissue sections and plant samples. Other parameters, represented by other or several different spectral regions, may provide the basis of statistical analyses of further specific histological properties in a whole set of samples.

### 2.2. Improving Image Contrast by Pattern Recognition

In order to increase specificity and contrast in the maps, we merged the spectral ranges used for chemical mapping and carried out multivariate analyses with the data. In general, such pattern recognition methods are extremely useful in the analysis of very different kinds of Raman data from plants [14,33,36,37], including maps of tissue sections [10,15,38], where, e.g., small variations in lignin composition within cell walls of different tissue regions can be identified [15].

**Figure 5:**
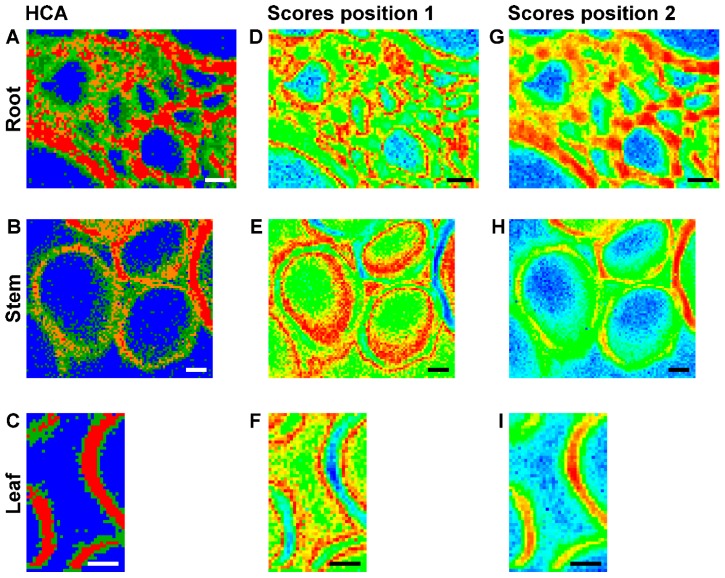
(**A**–**C**) Images using the class assignment of each spectrum as result of a hierarchical cluster analysis (HCA) with each respective mapping data set; (**D**–**I**) Maps based on the score value of the first principal component as a result of principal component analysis (PCA) of each respective mapping data set; (**D**–**F**) PC 1 scores map with color map using a scores value of zero as reference. (**G**–**I**) PC 1 scores map with color map using the respective maximum scores value as reference. Maximum value in (**G**) 0.4 (cf. Figure 7D for the corresponding scores plot); in (**H**) 0.55; and in (**I**) 0.5. HCA and PCA are based on spectra of three merged spectral regions of vector normalized first derivatives: 1070–1108 cm^−1^, 1313–1358 cm^−1^, and 1550–1800 cm^−1^. (**A**,**D**,**G**) data set from root (cf. Figure 2, last column); (**B**,**E**,**H**) data set from stem (cf. Figure 3, last column); (**C**,**F**,**I**) data set from leaf (cf. Figure 4, first column). Scale bars: 10 µm, mapping step size: 1 µm, excitation wavelength: 532 nm, excitation intensity: 1.7 × 10^6^ W/cm^2^, accumulation time: 1 s.

**Figure 6:**
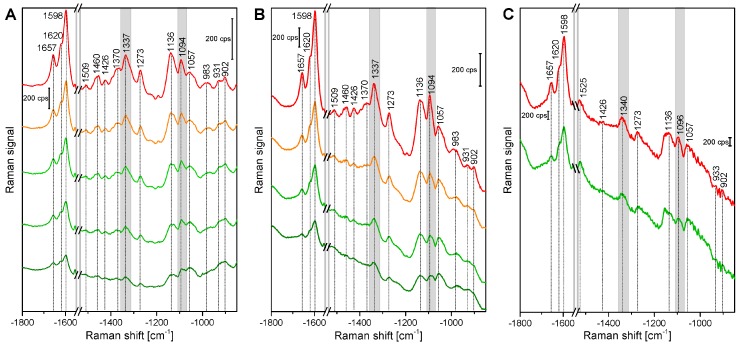
(**A**–**C**) Average spectra of the HCA clusters shown in Figure 5A–C with the same color codes of Raman maps of cross sections of (**A**) root; (**B**) stem; and (**C**) leaf tissue. HCA is based on spectra of three merged spectral regions of vector normalized first derivatives: 1070–1108 cm^−1^, 1313–1358 cm^−1^, and 1550–1800 cm^−1^.

Figure 5A–C a show false color images, based on the hierarchical cluster analysis (HCA) mapping data from the three data sets that were already displayed in Figure 2 (right column), Figure 3 (right column), and Figure 4 (left column), respectively. The HCA images were generated assuming different numbers of spectral groups that are formed by this unsupervised clustering approach. In all tissues, the areas of the lumina (Figure 5A–C, blue pixels) form one separate cluster, while the cell wall areas are subdivided. In the map of the root section shown in Figure 5A, the cell wall corners and middle lamellae can be separated (Figure 5A, red and orange pixels) from the secondary and surrounding cell walls (Figure 5A, green pixels). Examining the lignin marker bands at 1598 cm^−1^, 1520 cm^−1^, and 1657 cm^−1^ in the original spectra extracted from these regions of the maps, we find that they show higher relative intensities (see Figure 6) than in the other cluster spectra, indicating that these regions are enriched in both coniferyl aldehyde and coniferyl alcohol units. In fact, we find that the lignin density is highest in the cell corner regions, lower in the middle lamella, and lowest in secondary cell wall, in agreement with findings in other, non-related reports [39,40]. The result of the cluster analysis can also be explained by the differences with respect to the cellulose signals in the spectra. Cellulose is a crystalline array of many microfibrils, that is, parallel oriented chains that function as fundamental structural units. In the spectra corresponding to the secondary wall (green) regions in the map of Figure 5A, the orientation sensitive C-O-C cellulose band at 1093 cm^−1^ [8,29,30,31] appears a bit more prominent in the secondary cell wall than in the cell corners and middle lamella (Figure 5A red and orange pixels), indicating the higher amount/density of highly oriented microfibrils in this cell wall substructure.

As visible in the HCA maps of the stem (Figure 5B) and the leaf (Figure 5C) example, in most of the analyzed cell walls we cannot identify regions of corners and middle lamellae. The secondary cell walls in stem and leaf maps are clustered into two groups (Figure 5B, red and orange pixels) or form one class (Figure 5C, red pixels), while another cluster (Figure 5B,C, green pixels) also includes areas where the primary cell walls can be expected. Inspection of the spectra (not shown) revealed higher relative intensities of the lignin bands, pointing towards a higher degree of lignification in the orange/red regions.

Figure 5D–I shows maps that were reconstructed using the scores values of the first principal component (PC) obtained in a principal component analysis (PCA) on the same three merged spectral ranges as in the hierarchical cluster analyses. Each pixel in such an image represents the similarity with respect to other pixel spectra, and its corresponding spectrum is found at a specific position in the scores plot. The maps, based on the first PC that represents the highest variance in the data set, support the contrast and separation of cell wall substructures obtained by HCA mapping (Figure 5A–C). They separate the spectra of the cell corners and the middle lamellae in the root section (Figure 5D,G) and the spectra of the secondary cell walls in stem (Figure 5E,H) and leaf (Figure 5F,I).

**Figure 7:**
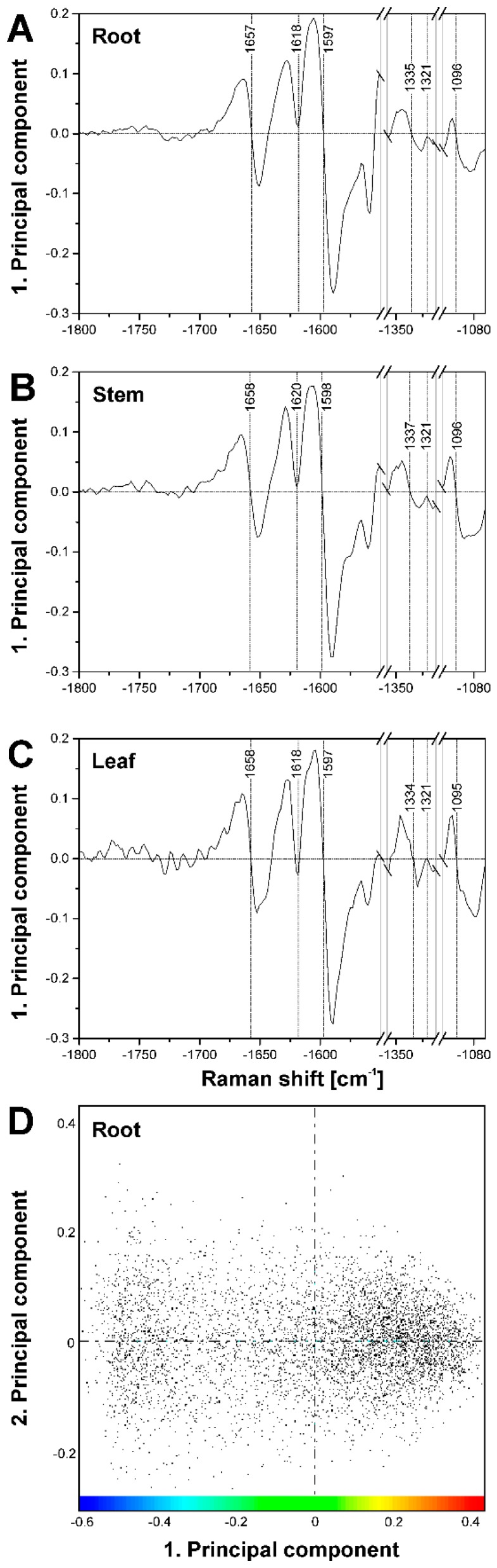
(**A**–**C**) Loadings of the respective first principal component corresponding to the PCA maps shown in Figure 5. (**A**) loading of PC1 for map shown in Figure 5D,G (root); (**B**) loading of PC1 for map shown in Figure 5E,H (stem); and (**C**) loading of PC1 for map shown in Figure 5F,I (leaf); (**D**) Scores plot corresponding to the loading in (**A**) and the PCA maps in Figure 5D,G. The color bar indicates the scale chosen for the PCA map in Figure 5G, using the maximum value of 0.4 as reference position.

As the corresponding loadings spectra of the first PC (Figure 7A–C) indicate, the main contributors to the image contrast in the three tissue types are the same, but small differences exist in the lignin signals, as well as in the shape of the cellulose bands. This is in agreement with the spectral differences discussed in the context of HCA cluster formation above.

Comparison of the three maps of Figure 5D–F and the respective corresponding maps of Figure 5G–I reveals that PCA imaging enables a ‘fine tuning’ of image contrast using the same data by fixing a particular reference position in the scores plot. The scores plot in Figure 7D shows the scores of the first two PCs from a PCA of the data set of Figure 5D,G. The color scale displayed in this scores plot along PC1 corresponds to the scale chosen to generate the map of Figure 5G. There, red colors indicate short distances to ‘lignin-like’ spectra that have positive scores values in PC1. Using this reference point (position 2), spectra from pixels colored in blue are very dissimilar and in this case represent the ‘lumen-like’ spectral pattern. For the images of Figure 5D–F, distances with respect to position 1, corresponding to a scores value of zero were used. This yields different contrast, emphasizing the regions that are quite dissimilar to both the cell corner regions and the lumen, corresponding to the green pixels in the HCA map (compare Figure 5D with Figure 5A).

### 2.3. Bleaching of Photosensitive Molecules

Interestingly, in some of the chemical maps of the stems and the leaves, the noisy regions in the images co-localize with regions of a high general signal, and the corresponding spectra show a high background, mainly caused by fluorescence (cf. last spectra in Figure 1). Figure 2F, Figure 3F and Figure 4F map the overall spectral intensity in the range 600–2000 cm^−1^, which provides information on such a background signal, and also highlights those spectra that have very high Raman signals. Therefore, also from these maps, the tissue morphology can be obtained, yet without many chemical specifics.

The autofluorescence of plant materials has successfully been used for the characterization of cell walls [30,35,41,42]. In the cell wall, several molecules such as lignin, cutin, suberin, or cellulose itself show autofluorescence, depending on the excitation wavelength [21,43]. Although it is often difficult to relate autofluorescence to one specific molecule, it can provide indications of the surroundings of the fluorescing species inside the cell wall or help a relative quantification of the lignification [35]. Furthermore, 2-photon fluorescence of lignin indicated the distribution of the molecule in the cell wall [15].

**Figure 8:**
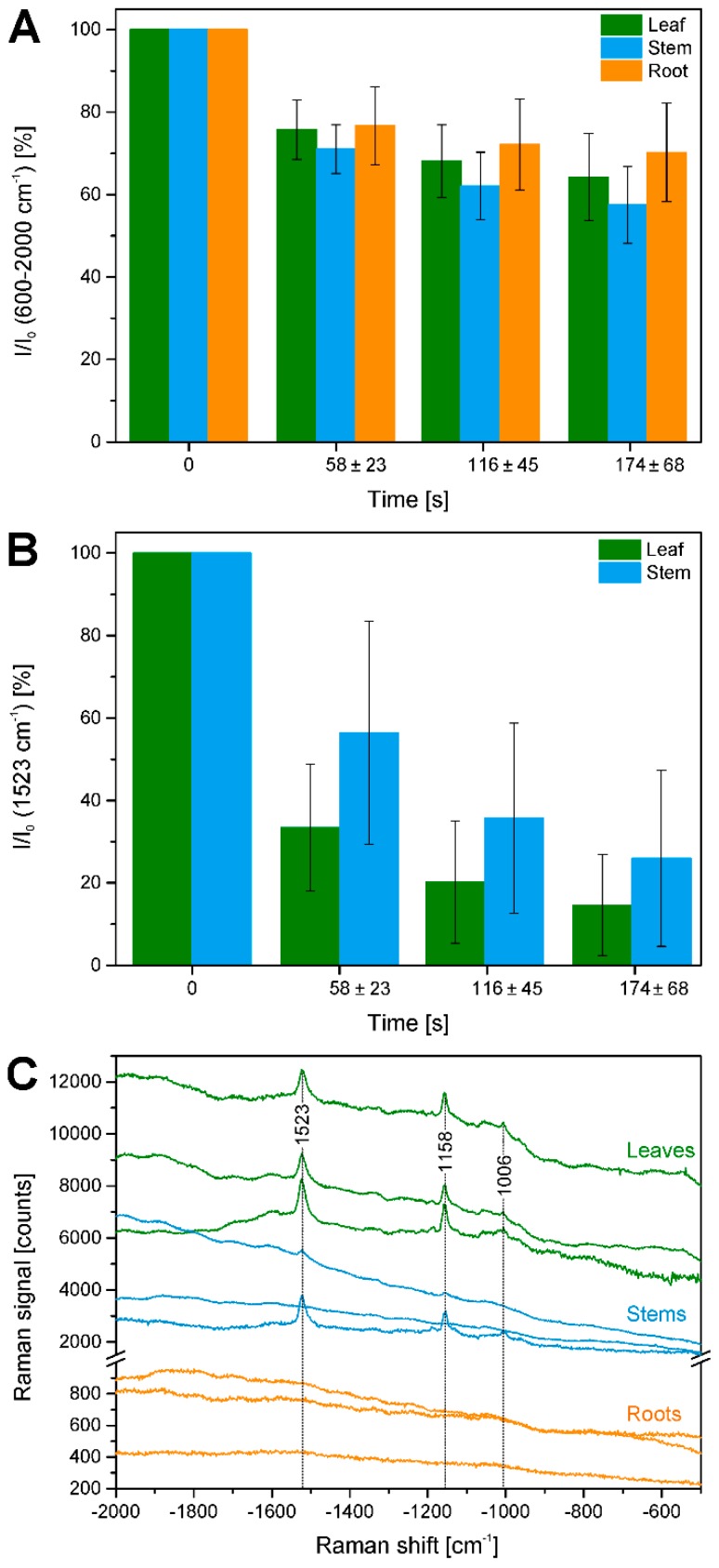
Quantification of bleaching effects. (**A**) Intensity in the region 600–2000 cm^−1^ (as measure of fluorescence background) integrated over a respective row of data points relative to the integrated intensity in the first row (*I*_0_) in all data sets from leaves, stems, and roots (cf. Table 1). Average bleaching times are 58 ± 23 s for the second row, 116 ± 45 s for the third row, and 174 ± 68 s for the fourth row; (**B**) Integrated intensity of the Raman band of carotenoid species at 1523 cm^−1^ over a respective row of data points relative to the integrated intensity in the first row (*I*_0_) in all data sets from leaves and stems (cf. Table 1). Average bleaching times are 58 ± 23 s for the second row, 116 ± 45 s for the third row, and 174 ± 68 s for the fourth row; (**C**) Difference spectra between the averages of the spectra of the first and the second row of the example maps shown in Figure 2, Figure 3 and Figure 4. To demonstrate absolute background signals, spectra are not stacked.

In the maps of Figure 3E and Figure 4E, very high overall signals are visible in the first few rows of each raster scan. Acquisition of Raman spectra (1 s per spectrum using an excitation intensity of 1.7 × 10^6^ W/cm^2^) starts in the bottom left corner of the map, moving line-wise to the top. Later, the background signal decreases with increasing mapping time. Figure 8A shows this effect for all data in the different plant organs and indicates that in all tissues bleaching of the background occurs. Bleaching of tissue autofluorescence has been described [28,44], and often may not be discussed in Raman studies of plant samples because the process occurs relatively fast and is not visible when several spectra of the same position are averaged or whole tissue areas are measured multiple times [7,16,17]. In the maps shown in Figure 2, Figure 3 and Figure 4, bleaching of the autofluorescence background serves us to a certain advantage, as it facilitates Raman imaging based on the remaining, non-bleached tissue constituents.

Nevertheless, as consequence of the photobleaching, the Raman spectra cannot show contributions from the destroyed molecules. As the analysis of the cellulose and lignin contributions discussed above, as well as other studies conducted at this excitation wavelength [15] demonstrated, the signal from these biomacromolecules enables detailed structural /vibrational characterization of the cell walls in spite of the selectivity in favor of the non-bleachable molecules. In fact, as comparison of NIR (785 nm)-excited and 532 nm-excited Raman spectra of similar samples showed [15], imaging and characterization of cell walls benefits greatly from the high scattering cross sections at the visible wavelength rather than from the absence of the bleaching effects in the NIR.

Figure 8B illustrates that also Raman signals undergo bleaching, in the case of the pre-resonant excitation of the carotenoid species during the raster scanning, using the band intensity at 1523 cm^−1^ of the C=C (ν_1_) stretching vibration of carotene. Due to the differential localization of carotenes in in the three plant tissues, the effect is observed in leaves and stems, and more pronounced in the leaves. A ratio was generated from the accumulated carotene intensities *I* in different segments of the map that correspond to different irradiation, that is, bleaching times and the intensity before the bleaching *I*_0_. Furthermore, difference spectra were calculated from the average spectra of the respective first and second row in each of the maps shown in Figure 2E, Figure 3E and Figure 4E (Figure 8C). As visible in the spectra of Figure 8C, the differences calculated from the leaves and stem maps show very distinct features of carotenoid species. 

The main tasks of the carotenoids in the photosynthetically active organs of the plant are the absorption of light and protection against oxygen radicals that may occur during photosynthesis. Chromoplasts containing carotenoids can get cut during the preparation of plant sections, and carotenoid molecules are removed during typical sample preparations that involve resins and solvents. Here, preparation without embedding media and storage in water enabled the carotenoids to adhere to some cell walls, where they contribute to the spectra (cf. Figure 1). Carotenoids in cucumber plants are mainly beta-carotene and lutein, and also include neoxanthin, violaxanthin, antheraxanthin, and zeaxanthin [45].

Taking into account the great instability of carotenoid molecules, the Raman spectral information obtained in situ during the photodepletion process is clearly of advantage for several reasons. Although the spectral signatures of the carotenoid pigments were discussed as main reason for the misclassification of Raman spectra of plant [1] and bacteria [19], other studies, where such depletion difference spectra were deliberately generated, showed that the pure carotenoid spectral features that are obtained can serve a variety of purposes [18]: Since the difference spectra give information about the in situ bleached carotenoid spectrum, they reveal carotenoid structure and composition while the molecules are embedded in their original biological matrix. In contrast, purification of carotenoid species from the plant tissues may change their excitation profile significantly. Furthermore, no carotenoid molecule is lost un-analyzed. Most importantly in the context of plant tissue Raman imaging, depletion difference carotenoid spectral fingerprints become available that may be highly specific for a specific tissue type and histological region, and that could serve in an efficient correction of the spectra from the same or other Raman mapping data. Such correction approaches have been proposed for other, non-imaging, Raman data from pollen and bacteria [18,19]. Thereby, in future applications, image contrast could be further improved by exploiting these and maybe other depletion difference spectra.

## 3. Materials and Methods

### 3.1. Sample Preparation

Plants of *Cucumis sativus* (Cucumber) were cultivated in potting soil (Einheitserde classic ED 73) in greenhouses at 22 °C, 16 h day/8 h night, and watered with tap water. Four cucumber plants, ranging in age from 11 days to 31 days were prepared.

Before sectioning the roots were washed thoroughly with water. For stem cross-sections, the hypocotyl and for leaf sections the central axis of the oldest true leaves (not the cotyledons) were taken and wiped clean with a moist, lint-free tissue. All plant materials were cut into ~2 cm pieces with razor blades and stored in water at 4 °C in the dark until microsectioning. Sectioning was carried out in water and without embedding using a vibratome (Microm HM 650 V, Walldorf, Germany). Stem transversal cross sections were cut with a thickness of 70 µm, root and leaf sections were cut at a thickness of 50 µm. The sections were stored in water at 4 °C in the dark until Raman experiments. The sections were placed on CaF_2_ slides with a droplet of water and sealed with coverslips.

### 3.2. Raman Measurements

The Raman spectra of leaf, stem and root sections were obtained using an imaging spectrometer couple to a microscope, by scanning the tissue placed on an x, y stage in 1 µm steps (microscope objective UPlanFLN 40×, NA 0.75, Olympus, Hamburg, Germany). For detection, a 1200 lines/mm grating with a liquid nitrogen cooled CCD detector (Symphony II CCD, Horiba, Munich, Germany) was used. The spectra were excited with a CW laser at 532 nm and a laser power of 10 mW (corresponding to an intensity of 1.7 × 10^6^ W/cm^2^). Raman scattering was collected with confocal illumination, spectra were accumulated for 1 s. Spectral resolution was 3–4 cm^−1^ in the full spectral range. Bright field micrographs were taken and adjusted in contrast.

### 3.3. Data pre-Processing and Analysis

Each spectrum was frequency calibrated using the spectrum of toluene, and spikes were removed using MATLAB (The MathWorks, Inc., Natick, MA, USA). All spectra and Raman maps were analysed using CytoSpec (CytoSpec, Berlin, Germany), and Origin (OriginLab, Northampton, MA, USA) software. Color scale bars for chemical images from the Raman data range from blue (low signal) to red (high signal).

Hierarchical cluster analysis (HCA) and principal component analysis (PCA) were carried out on vector normalized first derivatives of the Raman spectra using three spectral regions: 1070–1108 cm^−1^, mainly assigned to contributions from the C-C and C-O) vibrations of cellulose, 1313–1358 cm^−1^, assigned to deformation vibrations of cellulose (δ(HCC and δ(HCO)), and 1550–1800 cm^−1^, comprising mainly contributions from lignin. 

Distances are Euclidean distances, Ward’s algorithm was used for clustering. Before calculation of derivatives, Savitzky-Golay filtering was applied for smoothing by a 5-point quadratic polynomial.

HCA maps were constructed by assigning each class a color and combining it with the spatial coordinate of each spectrum. The number of classes was evaluated in an iterative process involving the comparison of class average spectra. In the typical maps, this resulted in the occurrence of two to five spectral classes. PCA maps were generated using the scores values of the first principal component.

## 4. Conclusions

In conclusion, we have demonstrated different possibilities for the generation of Raman images from sections of native, unembedded root, stem, and leaf tissues of cucumber plants and use the Raman maps to analyze morphological information. Univariate (chemical) images of the sections using contributions from the main cell wall components lignin and cellulose reveal substructures of the cell walls in the xylem tissue. Using multivariate analyses, including hierarchical cluster analysis and principal component analysis, the cell wall substructures can be visualized more clearly. Excitation of the Raman maps leads to bleaching of photosensitive tissue constituents, which could be harnessed for imaging using difference depletion methods in the future.

## Figures and Tables

**Table 1:** Overview over the data sets and histological parameters extracted from the Raman imaging data.

Plant Organ	Number of Maps	Number of Different Cells ^a^	Number of Complete Cells ^b^	Extracted Cell Wall Spectra ^c^	Cell Wall Spectra per Complete Cell	Mean Cell Wall Area per Map [%]
Roots	20	187	134	39,262	294	67 ± 13
Stems	18	112	56	30,282	538	52 ± 13
Leaves	17	71	42	24,552	592	44 ± 17

^a^ Different xylem cells contained in all sections investigated by Raman mapping. Also partially investigated cell walls were counted; ^b^ Partially investigated cells were counted as parts of a complete cell and summed up; ^c^ Determined as cell wall spectra by automated separation by HCA clustering.

**Table 2:** Band assignments in the Raman spectra of the data sets from tissue sections of cucumber root, stem, and leaves according to refs [3,8,22,23,24,25,26,27].

Band Position [cm^−1^]	Tentative Assignment to Molecule
901	δ(HCC) and δ(HCO) at C-6, cellulose
927	ω(CCH), lignin;
983	ν(CC) and ν(CO), cellulose
1005	ν_3_ Methyl rocking, carotenoid
1059	ν(CC) and ν(CO), cellulose
1092	ν(CC) and ν(CO), cellulose
1136	ν(CC) and ν(CO), cellulose
1156	ν_2_ C-C, carotenoid
1271	Aryl-O of Aryl-OH and Aryl-O-CH_3_; guaiacylring mode (with CO-group)), lignin
1337	δ(HCC) and δ(HCO) cellulose
1376	δ(HCC) and δ(HCO), and δ(HOC), cellulose
1426	δ(O-CH_3_), δ(CH_2_), guaiacyl ring, lignin
1461	δ(O-CH_3_), CH_2_ scissoring, guajacyl ring (with C=O group), lignin; δ(HCH) and δ(HOC), cellulose
1509	ν_as_(Aryl ring), lignin
1523	ν_1_ C=C, carotenoid
1598	ν_s_(Aryl-Ring), lignin
1621	ν_conj_.(Ring C=C) of coniferylaldehyde, lignin
1658	ν_conj_.(Ring C=C) of coniferylalcohol, ν(C=O) of coniferylaldehyde, lignin

Abbreviations: ν, stretching vibration; δ, deformation vibration; ω, wagging; s, symmetric; as, antisymmetric; conj., conjugated.
